# Dupilumab Effectiveness in Patients with Severe Allergic Asthma Non-Responsive to Omalizumab

**DOI:** 10.3390/jpm15020043

**Published:** 2025-01-23

**Authors:** Diego Bagnasco, Benedetta Bondi, Luisa Brussino, Stefania Nicola, Paolo Cameli, Angelica Tiotiu, Giuseppe Guida, Chiara Gollinucci, Dina Visca, Antonio Spanevello, Laura Pini, Marco Caminati, Gianenrico Senna, Cristiano Caruso, Rikki Frank Canevari, Melania Bertolini, Sara Fedele, Marcello Mincarini, Giorgio Walter Canonica, Fulvio Braido, on behalf of the SANI group

**Affiliations:** 1 Respiratory and Allergy Clinic, IRCCS Ospedale Policlinico San Martino, 16132 Genoa, Italy; diego.bagnasco@dimi.unige.it (D.B.); marcello.mincarini@hsanmartino.it (M.M.); fulvio.braido@unige.it (F.B.); 2 Department of Internal Medicine (DIMI), University of Genoa, 16132 Genoa, Italy; 3 SCDU Immunology and Allergology, AO Ordine Mauriziano, 10128 Turin, Italy; luisa.brussino@unito.it (L.B.); stefania.nicola@outlook.com (S.N.); 4 Respiratory Diseases Unit, Department of Medicine, Surgery and Neurosciences, University of Siena, 53100 Siena, Italy; paolo.cameli@unisi.it; 5 Department of Pneumology, University Hospital Saint-Luc, 1200 Brussels, Belgium; iuliana.tiotiu-cepuc@saintluc.uclouvain.be; 6 Pole Pneumology, ENT, and Dermatology-LUNS, Institute of Experimental and Clinical Research (IREC), UCLouvain, 1200 Brussels, Belgium; 7 Severe Asthma and Rare Lung Disease Unit, San Luigi Gonzaga University Hospital, Orbassano, 10043 Turin, Italy; giuseppe.guida@unito.it (G.G.); chiara.gollinucci@unito.it (C.G.); 8 Department of Clinical and Biological Sciences, University of Turin, Orbassano, 10043 Turin, Italy; 9 Division of Pulmonary Rehabilitation, Istituti Clinici Scientifici Maugeri, IRCCS, 21049 Tradate, Italy; dina.visca@icsmaugeri.it (D.V.); antonio.spanevello@icsmaugeri.it (A.S.); 10 Department of Medicine and Surgery, Respiratory Diseases, University of Insubria, 21100 Varese, Italy; 11 Respiratory Medicine Unit, ASST-Spedali Civili, 25123 Brescia, Italy; laura.pini@unibs.it; 12 Department of Clinical and Experimental Sciences, University of Brescia, 25122 Brescia, Italy; 13 Department of Medicine, University of Verona, 37129 Verona, Italy; marco.caminati@univr.it (M.C.); gianenrico.senna@univr.it (G.S.); 14 UOSD Allergology and Clinical Immunology Unit, Fondazione Policlinico Universitario “A. Gemelli” IRCCS, Università Cattolica del Sacro Cuore, 00168 Rome, Italy; cristiano.caruso@unicatt.it; 15 ENT Department, IRCCS Policlinico San Martino, University of Genoa, 16132 Genoa, Italy; canevari@edu.unige.it (R.F.C.); melania.greta.bertolini@gmail.com (M.B.); 16 Department of Biomedical Sciences, Humanitas University, 20072 Pieve Emanuele, Italy; giorgio_walter.canonica@hunimed.eu; 17 Personalized Medicine, Asthma and Allergy, Humanitas Clinical and Research Center IRCCS, 20089 Rozzano, Italy

**Keywords:** severe asthma, nasal polyps, CRSwNP, dupilumab, omalizumab, clinical remission, anti-IL-4, anti-IgE, switch, biologics

## Abstract

**Background/Objectives**: Severe allergic asthma is usually treated with omalizumab; however, this drug may not be effective for every patient. By its action, dupilumab could be an alternative in these patients. The objective of this study was to evaluate the efficacy of dupilumab in patients with severe allergic asthma, non-responsive to omalizumab, according to the maintenance of their oral corticosteroid (OCS) dose, an exacerbation rate decrease, or poor control of the disease, despite optimized treatment. **Methods**: A retrospective analysis of data from severe asthma clinics was performed, observing the efficacy of the switch to dupilumab in patients who experienced a failed treatment with omalizumab. **Results**: Forty-two patients were included. Dupilumab proved to be effective in patients who experienced a failed omalizumab treatment, with a significant reduction in the exacerbation number and OCS use. Furthermore, remission of the disease, according to the Severe Asthma Network of Italy (SANI) criteria, was achieved in 35 patients, with complete remission in 19 (45%) and partial remission in 16 (38%). The analysis of the predictors of the success of dupilumab therapy in achieving clinical remission, through univariate analysis of the data at baseline, showed that complete remission was more easily reached in patients with concomitant aspirin (ASA) intolerance or in those with nasal polyposis. **Conclusions**: Dupilumab is an effective drug for the treatment of patients with severe asthma with an allergic component, with better benefits in patients with an ASA intolerance or nasal polyposis.

## 1. Introduction

Asthma is a chronic respiratory disease characterized by airway inflammation, hyperresponsiveness, and episodic airflow obstruction [1,2]. Affecting approximately 300 million people worldwide, asthma is a significant public health burden, with allergic asthma representing a greater percentage of patients [3].

Despite advances in asthma management, the development of new drugs and associations, and the major use of triple therapy (inhaled corticosteroids—ICSs; long-acting beta 2 agonists—LABAs; and a long-acting muscarinic antagonist—LAMA) [4] for achieving control of the disease, a subgroup of patients experiences persistent symptoms and frequent exacerbations, necessitating the use of oral corticosteroids (OCSs). To reduce the use of OCSs and, consequently, their side effects due to short and prolonged cycles [5,6], biological drugs have been studied, developed, and marketed. In order of time, the first marketed was omalizumab, a monoclonal antibody targeting immunoglobulin E (IgE), and it became the principal treatment option for patients with severe allergic asthma.

Omalizumab’s mechanism of action is linked to the binding of circulating IgE and, consequently, the ability to reduce its availability to interact with other cells such as eosinophils, mast cells, and basophils, thereby mitigating the allergic cascade and decreasing airway inflammation.

Since its approval, omalizumab has been shown to reduce asthma exacerbations, improve lung function, and enhance the quality of life in patients with moderate to severe allergic asthma [7]. Clinical trials [8,9] and real-world studies [10,11,12,13] have demonstrated its efficacy and safety [14], solidifying its role as an add-on therapy for patients who remain symptomatic, despite the highest dose of ICSs/LABAs with or without LAMA introduction [15].

However, despite its proven efficacy, omalizumab is not completely effective for all patients, and several of them may experience poor control of their symptoms, adverse reactions [16], or a diminished response over time.

Another critical point of omalizumab use is its dosing. The required dose varies based on a patient’s weight and total IgE levels, often leading to multiple injections at once, with up to four every two weeks. This factor may make omalizumab more difficult to manage than other drugs.

This has led to a growing interest in exploring alternative targeted therapies, especially those with different mechanisms of action.

Among the mechanisms of greatest interest is that of dupilumab, an antibody directed to the interleukin (IL)-4 receptor alpha (IL-4Rα), similar to IL-13. If blocked, it can act at both the level of the more typical eosinophilic inflammatory cascade and on the allergy-related cascade of B cells, IgE, and basophils [17,18,19,20].

Dupilumab has demonstrated its efficacy in clinical trials for severe asthma, particularly in patients with elevated biomarkers of type 2 (T2) inflammation, such as eosinophils and fraction of exhaled nitric oxide (FeNO) [21,22,23]. Its approval has provided a new therapeutic opportunity for asthma patients, encompassing both treatment-naïve individuals and those who have not achieved satisfactory outcomes with omalizumab or who have experienced adverse effects from anti-IgE agents due to their proven mechanism on the allergic inflammation pathway [18,23].

The main objective of this study was to evaluate the efficacy of switching from omalizumab to dupilumab in severely asthmatic patients with an allergic phenotype, who are non-responsive to anti-IgE therapy, in terms of an exacerbation rate reduction, a decrease in OCS use, and improved lung function, as well as in those with uncontrolled asthma, despite optimized and maximal treatment. The efficacy of the switch was evaluated by the reduction in the exacerbation rate and OCS intake; the improvement in lung function parameters, which was estimated from the forced expiratory volume per second (FEV1); a decrease in T2 inflammation biomarkers (e.g., eosinophils, IgE, and FeNO); and an improvement in asthma control, as measured by the asthma control test (ACT). For the OCS use, we calculated the dose taken in one year, measured in grams of prednisone (g/y), which is equivalent to the sum of the amount taken during cycles, the amount prescribed to manage exacerbations, and the fixed dose in OCS-dependent patients.

As secondary endpoints, we evaluated the impact on nasal symptoms, measured with the sino-nasal outcome test (SNOT-22) in patients with nasal polyps as a comorbidity of asthma (both studied biologics can be prescribed for chronic rhinosinusitis with nasal polyps—CRSwNP) and through the identification of patient characteristics that may predict clinical remission after one year of treatment with dupilumab, with the aim of helping physicians choose the most suitable biologic for each specific patient.

## 2. Materials and Methods

Data from nine severe asthma centers, eight from Italy (Genoa, Siena, Turin 2, Brescia, Tradate, Rome, Verona) and one from France (Nancy), were collected. The only inclusion criterion was the use of dupilumab in patients previously treated with omalizumab and switched to the anti-IL-4R due to poor efficacy, as judged by clinicians based on the exacerbation rate, OCS use, and ACT score. No patients were selected or excluded from the analysis. All patients fit the criteria of being severely asthmatic, according to the ATS/ERS guidelines [24]. Baseline data were collected at the time of switching from omalizumab to dupilumab for all patients after a washout period of at least 28 days. A second data collection point occurred 12 months after starting dupilumab treatment. The prescribing criteria for both drugs were either those of the Italian or France national registry. Before administration of any biologic, the patients were required to have spirometry, the measurement of FeNO, a complete blood count (CBC) for eosinophil and IgE count, as well as completion of the ACT and SNOT22 questionnaire. These examinations were also performed at the time of the switch between omalizumab and dupilumab and at one year after the start of dupilumab therapy itself. The other demographic and clinical data investigated are shown in Table 1.

The study was conducted in accordance with the Declaration of Helsinki and approved by the Institutional Review Board of Genoa (approval code: EC 2017, ID 3663, 1 January 2017 for Italian data) and by the Institutional Review Board of CEPRO (approval code: 2017-042, 27 November 2017 for French data).

For the concept of remission, we used the criteria according to the SANI registry, distinguishing between patients who achieved complete and partial remission [25]. These criteria involved the discontinuation of OCS use and, for partial remission, at least two of following: no exacerbations, FEV_1_ stabilization, and ACT ≥ 20, and all of them for complete clinical remission [26]. The proportion of patients who achieved complete and partial clinical remission, after 1 year of treatment with dupilumab, was evaluated. Remission was not examined in the omalizumab treatment phase, as only those patients who did not respond to omalizumab were included in the study, and this would have resulted in significant bias in the remission results.

### Statistical Analysis

Data were expressed as the mean and standard deviation (SD) or the absolute number and percentage (%). The statistical analyses used were the Student’s *t*-test, exact Fisher’s test, and univariate analysis, when necessary, using the statistical program Jamovi^®^ (version 2.3.28).

## 3. Results

### 3.1. General Sample

Data about 42 patients were collected and analyzed; these patients had a mean age of 55 ± 12 years (range 26–83), 23 (55%) of whom were female, with a mean age of onset of 24 ± 17 years for the whole sample. Allergic status was evaluated in all patients, with 40 of them (95%) allergic to perennial allergens, principally house dust mites (HDM) (n = 30, 71%) and mold (n = 8, 19%), followed by pets (dog and cats) and pellitory, which is perennial both in Italy and France. Data about the presence of aspirin-exacerbated respiratory disease (AERD) were already measured and found to be present in ten (24%) of the observed patients. Bronchiectasis was present in six (14%) patients. Body mass index (BMI) resulted stable during the period of observation, with a mean of 26 ± 4 kg/m2. In terms of the T2 biomarkers, all patients were sensitized to a perennial allergen, where 31 (74%) had a blood eosinophils value > 150 cells/mcl and 17 (40%) a FeNO > 20 ppb. (Table 1).

Before switching to dupilumab, the mean duration of omalizumab administration was 42 ± 31 months with a median dose of 300 mg monthly.

### 3.2. Asthma Outcomes During the Omalizumab Administration

The effect of omalizumab administration was only observed in the reduction in the number of exacerbations (4.00 ± 2.45 to 2.60 ± 2.69; *p* = 0.015) and hospitalizations (0.4 ± 0.8 to 0.1 ± 0.3; *p* = 0.029). There was no effect on OCS dose reduction, measured in g/year (2.86 ± 4.3 to 2.24 ± 2.67; *p* = 0.43), nor did it modify the percentage of patients who reduced their steroid dependence, which remained unchanged at 16 (38%) in the total sample. An improvement in the FEV1 was evidenced, although this time, it was not statistically significant (70 ± 23 vs. 79 ± 22; *p* = 0.084). The ACT score also improved by two points from 15 ± 5 to 17 ± 5 (*p* = 0.056) and did not reach statistical significance or minimal clinically important differences (MCID). A partial effect on SNOT-22 was demonstrated with no statistical significance (51 ± 14 vs. 43 ± 24; *p* = 0.337). At the time of the switch, despite a reduction in the annual dose, only two (5%) patients interrupted their OCS intake, another two (5%) did not have exacerbations, twenty-two (66%) improved their ACT more than 20 points, and twenty-three (55%) maintained a stable FEV1 value. Nevertheless, only two patients achieved a partial SANI remission of symptoms, but none of them reached a sufficient ACT score (mean 14 points) to be considered controlled, whereby they would be switched. No significant reduction in the T2 inflammation biomarkers was found after treatment with omalizumab (Table 1).

### 3.3. Asthma Outcomes After Switching to Dupilumab

**Figure 1:**
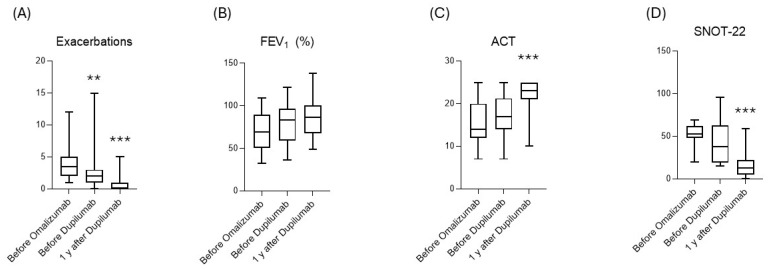
Trend of main outcome at baseline, after omalizumab, and after dupilumab. (**A**) Exacerbations’ variation; (**B**) FEV_1_ (%) variation; (**C**) ACT value; (**D**) SNOT-22 in patients with concomitant CRSwNP. FEV1%: % of predicted of forced expiratory volume in 1 s; ACT: asthma control test; SNOT-22: sino-nasal outcome test; CRSwNP: chronic rhinosinusitis with nasal polyps. *** *p* < 0.01. ** *p* > 0.05.

**Figure 2:**
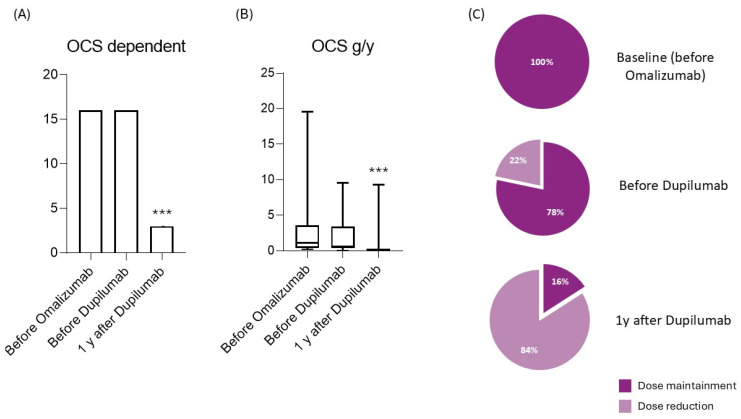
OCS intake trend over observation time. (**A**) OCS-dependent patients at checkpoint time; (**B**) OCS intake, measured in g/y; (**C**) percentage of patients able to reduce dose of OCS. *** *p* < 0.01.

After one year of dupilumab, we observed a significant decrease in the exacerbation rate (2.60 ± 2.69 to 0.71 ± 1.25; *p* < 0.001) and hospitalizations (0.3 ± 0.03 to 0 ± 0.22; *p* = 0.005). In contrast to the previous period, we also observed a significant decrease in the number of OCS-dependent patients (from 16 to 3; *p* = 0.001) and in the use of oral corticosteroids (g/y) in the entire analyzed sample (from 2.24 ± 2.67 g to 0.45 ± 1.48 g; *p* = 0.001). As far as lung function is concerned, the mean value of the patients reached and exceeded the value of 80% (28 patients, corresponding to 67% of the sample) despite the lack of statistical significance. The ACT score increased by 5 points after 12 months of administration (17 ± 5 vs. 22 ± 4; *p* < 0.001); a significant improvement was also seen in nasal symptoms, assessed with the SNOT-22 (24 ± 24 vs. 17 ± 15 points; *p* < 0.001), with both values overcoming the MCID level. Interesting observations were also made on the drug’s effects on the serum IgE level, the specific target of omalizumab, which decreased significantly after 12 months of treatment with dupilumab (508 ± 419 vs. 133 ± 152; *p* = 0.009). (Table 1 and Figure 1 and Figure 2).

### 3.4. Remission with Dupilumab

**Figure 3:**
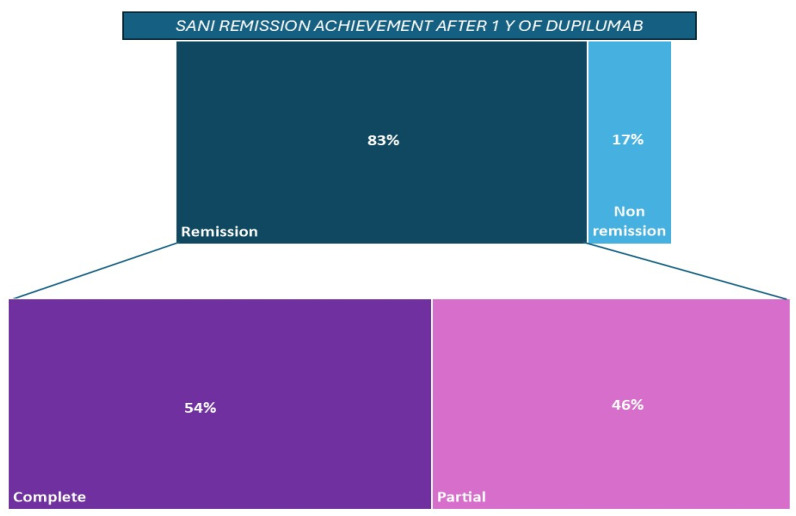
Achievement of clinical remission according to SANI criteria, with distribution in partial and complete remission. Patients in complete remission also achieved the partial remission criteria.

The concept of remission, as previously described, was differentiated into complete and partial remission, according to the SANI definition. Partial remission was achieved by 35 (83%) patients (group includes patients in complete remission), and 19 (45%) achieved complete remission; only 7 (17%) patients did not achieve remission with dupilumab treatment. (Table 2 and Figure 3).

### 3.5. Univariate Analysis

According to the univariate analysis performed on the baseline values to predict remission elements, before omalizumab treatment, the presence of aspirin-exacerbated respiratory disease (AERD) (OR 7.64; CI 1.38–42.3; *p* = 0.02) and concomitant CRSwNP (OR 4.09; CI 1.04–16.2; *p* = 0.04) were found to be positive predictors for complete remission after dupilumab treatment (Table 3).

### 3.6. Biomarkers

**Figure 4:**
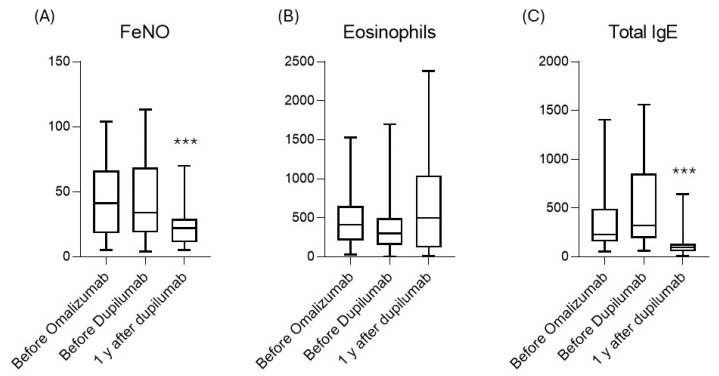
Variations at checkpoints of main biomarkers of T2 inflammation: (**A**) FeNO; (**B**) Eosinophils; (**C**) Total IgE. *** *p* < 0.01.

In terms of the biomarkers of type 2 inflammation, during the administration of omalizumab, there was no reduction in FeNO, eosinophils, or IgE counts. After switching to dupilumab, we saw a reduction in FeNO (44 ± 32 ppb vs. 24 ± 17 ppb; *p* = 0.002) and in the total IgE count (508 ± 419 kU/L vs. 133 ± 152 kU/L; *p* = 0.009) (Table 1, Figure 4).

## 4. Discussion

The efficacy of omalizumab, in the treatment of patients with severe allergic asthma, has now been largely proven, both in clinical studies [8,15] and in everyday practice [12,27,28]. The safety of the drug, demonstrated by the possibility of omalizumab administration in pregnancy [29,30,31] and by the few adverse events recorded during the pharmacovigilance phases [14], has made omalizumab a leading product in the treatment of patients with allergic asthma. This drug has also been tested and marketed in nasal polyposis [32,33,34,35,36], where it has shown efficacy in reducing the impact of polyps, both as a single disease and as a comorbidity of severe asthma.

A comprehensive understanding of the mechanisms sustaining asthma inflammations, along with the development of new mediators involved in the asthma inflammation, allow the production of different drugs, targeting cytokines or receptors, able to control the disease while being OCS sparing. In recent years, various products have been developed and subsequently marketed, with the aim of controlling asthma, through different mechanisms beyond IgE inhibitions. One proposed mechanism involves the interaction of dupilumab with IL-4. The binding of IL-4r, among its various effects, has the role of modulating the inflammatory response associated with B cells, IgE, and consequently, the cascade of events triggered by allergens [19].

The process begins when IL-4 binds to its receptor, IL-4R, on the surface of B cells. This interaction triggers a cascade of intracellular signaling pathways, primarily the Janus kinase (JAK)-signal transducer and activator of transcription 6 (STAT6) pathway. Activation of STAT6 leads to its translocation into the nucleus, where it promotes the transcription of specific genes that drive B cell differentiation and class switching to IgE production. Furthermore, this process is facilitated by the enzyme activation-induced cytidine deaminase (AID), which is upregulated in the presence of IL-4. As a result, B cells produce IgE, which subsequently binds to high-affinity Fc epsilon receptors (FcεRI) present on the surface of mast cells and basophils, sensitizing them for future encounters with specific antigens. Thus, in the event of interaction between dupilumab and IL-4R, all these mechanisms are downregulated, making dupilumab a potential alternative to omalizumab in patients with allergic IgE-mediated asthma [37,38].

Given the availability of dupilumab as an alternative therapy for severe allergic asthmatic patients, there is a growing interest in understanding the clinical efficacy associated with the administration of the anti-IL-4R both in biologic-naïve patients and, more interestingly, in one switching from omalizumab, who, despite being allergic, did not respond adequately to that treatment. Factors such as efficacy, safety, tolerability, and patient-reported outcomes are certainly of particular interest in evaluating this transition.

The possibility of acting, with a single drug, on multiple mechanisms, is crucial for more than one reason. First, data from the literature, show that patients, very often, do not have a clearly defined endotype, but multiple disease triggers are present simultaneously. For instance, a recent study by Denton et al. [39], shows how high values of eosinophils, high values of FeNO, and an allergic component frequently coexist in the same subject. Thus, the possibility to act on these patients with a drug capable of interacting simultaneously with all these components may be of added value, offering significant advantages compared to drugs targeting a single component. In addition, the aforementioned article shows that only 12% of the 1175 patients analyzed did not express any of the above-described markers. In our study cohort, while all patients exhibited a positive test for a perennial allergen (74%), a substantial proportion also had an elevated peripheral eosinophilic count ≥ 150 cells/mcl and elevated FeNO ≥ 20 ppb (40%), highlighting the heterogeneity of disease mechanisms even within our study population.

The heterogeneity of inflammatory mechanisms in asthma, as previously suggested by other authors in observational studies and post-hoc trials [40], may explain the lack of response to anti-IgE in our patient cohort. Indeed, it is well known how type 2 and non-type 2 inflammation can be triggered by multiple factors, including, of course, allergic ones, as well as airway infections or mechanisms of epithelial damage [41]. Consequently, the ability to target more than one factor results in added value, providing clinical benefits.

A noteworthy observation from the study pertains to the two drugs’ impact on biological markers. We observe that the patients, who were non-responsive to omalizumab, maintained statistically unchanged levels of FeNO and total IgE during the administration of that drug; after the switch to dupilumab, a reduction in both biomarkers was observed (Figure 3). The FeNO values in patients with severe asthma correlate with the disease control and exacerbation risk, with a direct statistical proportion; as the nitric oxide values increase, the risk of exacerbation increases too [42,43]. Thus, reducing FeNO levels seems to decrease the risk of poor symptom control, an outcome achieved with dupilumab, as demonstrated by the reduction in the mean of exacerbations after switching to the anti-IL-4R. A similar observation can be made with the IgE values and the risk of exacerbations: reducing the total IgE values with biological drugs has been proven to decrease the risk of exacerbations [44]. Although omalizumab is able to bind this immunoglobulin, it does not effectively reduce their levels, unlike dupilumab.

From a clinical perspective, it is also interesting to evaluate the response on the nasal component, as measured by SNOT-22. Our data indicated that with the anti-IgE drug, the rhinosinusitis symptoms were not controlled, although omalizumab is a drug indicated both in asthmatic patients with nasal polyposis and in nasal polyposis without asthma [33]. After 12 months of therapy with dupilumab, we observed a statistically significant reduction in the SNOT-22 values, reaching the MCID for this parameter [45].

Upon univariate analysis, AERD and CRSwNP emerged as significant predictors of a favorable response to switching to dupilumab treatment, with the goal of achieving complete clinical remission. Real-life studies have shown that both omalizumab and dupilumab are effective in inducing tolerance to ASA in patients with AERD. Omalizumab has also been reported in the literature as an adjuvant desensitization drug for this disease [46], as a therapy that reduces respiratory symptoms in affected patients [47]. The observation that this comorbidity, at least in the cohort of patients analyzed, facilitates the efficacy of dupilumab, provides additional support for considering, even as a first-line drug, anti-IL-4r in this patient population. Other interesting evidence concerns CRSwNP, a comorbidity that appears to be a positive prognostic factor for the efficacy of dupilumab in patients with allergic asthma. Both drugs have been studied and marketed in asthma and CRSwNP, with evidence of efficacy either in trials or in real life. The data from this study add an additional piece, showing that disease remission is more easily achieved in comorbid asthma-CRSwNP patients when treated with dupilumab.

In addition, the new concept of disease remission [48,49,50,51,52,53] makes the criteria for defining a patient whose disease is fully controlled by a drug even more rigorous, requiring the physician to make a judicious choice about which patients to keep on therapy and who to treat with alternative strategies to achieve remission.

Regarding clinical remission, since patients were, by definition, not fully responsive to omalizumab, this measure was evaluated only at the phase of treatment with dupilumab. The definition provided by SANI allows two categories of patients, responders and partial responders, to be considered, providing a greater dynamic regarding the response to therapy [26].

After 1 year of dupilumab therapy, complete remission was achieved in 45% of patients observed, with partial remission in 38%. The innovativeness of the concept of partial remission allows evaluating the efficacy of the drug, even without the complete achievement of all remission parameters. Furthermore, as demonstrated in the literature, it is desirable that at least a proportion of patients in partial remission, when continuing the drug, achieve complete remission over time [25,54]. This complexity underscore the challenges faced by physicians when managing patients who do not achieve complete remission after one year, in deciding whether to continue the current therapy or possibly switch treatment, emphasizing the importance of precisely defining the patients’ phenotype before targeting for biological therapy and following them over time.

## 5. Conclusions

In conclusion we observed that, although the cohort studied was not very large, an undeniable efficacy of dupilumab in severely allergic asthmatic patients who did not benefit from omalizumab therapy. The clinical remission was obtained in a high percentage of patients, whether partial or complete. The data shown, therefore, urge patients with an allergic type of asthma to consider the best possible therapeutic approach, both in patients already treated with anti-IgE and in biological-naïve patients, in order to consider switching or even using dupilumab as a biological therapy, at the first instance.

## Figures and Tables

**Table 1:** Data of the studied cohort at baseline, after omalizumab treatment, and after dupilumab switch.

	Before Oma	Post Oma/Before Dupi	*p*-Value	Post 1y Dupi	*p*-Value
**Female**	23 (55%)				
**Age**	55 ± 12				
**Age onset**	24 ± 17				
**AERD**	10 (24%)				
**Sensibilization to perennial allergens**	40 (95%)				
HDM	30 (71%)				
Mold	8 (19%)				
**Atopic dermatitis**	3 (7%)				
**Bronchiectasis**	6 (14%)				
**Smoking status**					
Current	9 (21%)	4 (10%)	0.587	8 (19%)	0.865
Former	8 (19%)	8 (19%)	>0.999	8 (19%)	<0.999
Never	25 (60%)	30 (71%)	0.745	26 (62%)	0.743
**BMI**	26 ± 4	26 ± 4	>0.999	26 (4)	>0.999
**CRSwNP**	26 (62%)	26 (62%)	>0.999	20 (48%)	0.564
**Exacerbations**	4.0 ± 2.45	2.6 ± 2.7	0.015	0.71 ± 1.3	<0.0001
**Hospitalizations**	0.4 ± 0.8	0.1 ± 0.3	0.029	0 ± 0.22	0.005
**OCS dep patients**	16 (38%)	16 (38%)	0.047	3 (7%)	0.001
**OCS dose g/y**	2.9 ± 4.3	2.2 ± 2.7	0.434	0.45 ± 1.5	0.001
**FEV1 (L)**	2.19 ± 0.93	2.34 ± 2.7	0.463	2.56 ± 0.87	0.132
**FEV1 (%)**	70 ± 23	79 ± 22	0.084	86 ± 20	0.131
**Eosinophils**	516 ± 401	381 ± 338	0.116	626 ± 557	0.348
**IgE total**	372 ± 307	508 ± 419	0.160	133 ± 152	0.0085
**FeNO**	44 ± 29	44 ± 32	0.941	24 ± 17	0.0017
**ACT**	15 ± 5	17 ± 5	0.056	22 ± 4	<0.0001
**SNOT-22**	51 ± 14	43 ± 24	0.337	17 ± 15	<0.0001

BMI: body mass index, CRSwNP: chronic rhinosinusitis with nasal polyps; AERD: aspirin-exacerbated respiratory disease; OCS: oral corticosteroids; FEV1: forced expiratory volume in 1 s; FeNO: fractional exhaled nitric oxide; ACT: asthma control test; SNOT-22: sino-nasal outcome test.

**Table 2:** Patients in remission according to SANI criteria. * All patients in complete remission also fulfilled the partial remission criteria. § Required value for parameters: no exacerbations, FEV_1_ stabilization (improve, ≥80% or a decline lower than 150 mL from previous year), ACT ≥ 20.

Remission		Non Remission	
35 (83%)		7 (17%)	
**Remission category**			
Complete		Partial	
19 (45%)		16 (38%)^*^	
**Achieved criteria for remission ^§^**			
**Exacerbations**	**FEV1**		**ACT**
26 (62%)	36 (86%)		30 (71%)

**Table 3:** Univariate analysis of the predictors for complete clinical remission from the baseline (pre-omalizumab) data.

	OR	CI	*p*-Value
**Sex (M vs. F)**	1.73	0.51–0.91	0.38
**Age**	0.98	0.93–1.03	0.34
**Asthma onset**	0.99	0.96–1.04	0.94
**BMI**	0.94	0.79–1.11	0.94
**Smoke (y vs. n)**	0.74	0.05–1.93	0.69
**Packs/y**	0.96	0.89–1.03	0.23
**CRSwNP(y/n)**	4.09	1.04–16.2	0.04
**Sensitization to perennial allergen**	0.24	0.02–2.55	0.24
**Sensitization to seasonal allergen**	1.33	0.31–5.60	0.70
**Atopic dermatitis**	0.37	0.04–3.89	0.41
**Bronchiectasis**	0.56	0.09–3.45	0.53
**AERD**	7.64	1.38–42.3	0.02
**Exacerbations**	0.97	0.75–1.24	0.80
**Hospital admissions**	0.43	0.07–2.76	0.38
**OCS (g/y)**	1.00	1.00–1.00	0.21
**LAMA**	0.49	0.18–2.31	0.49
**Antihistamine**	0.83	0.30–4.59	1.17
**Montelukast**	1.50	0.37–6.14	0.57
**FEV1 (L)**	1.90	0.04–3.84	0.08
**FEV1 (%)**	1.01	0.99–1.04	0.38
**Eosinophils (cell/mcl)**	1.00	0.99–1.00	0.24
**Total IgE**	1.00	0.99–1.00	0.76
**FeNO**	1.02	0.98–1.04	0.32
**ACT**	1.07	0.93–1.22	0.34
**SNOT-22**	0.78	0.90–1.08	0.98

BMI: body mass index, CRSwNP: chronic rhinosinusitis with nasal polyps; AERD: aspirin-exacerbated respiratory disease; OCS: oral corticosteroids; LAMA: long-acting antimuscarinics; FEV1: forced expiratory volume at 1 s; FeNO: fractional exhaled nitric oxide; ACT: asthma control test; SNOT-22: sino-nasal outcome test.

## Data Availability

The original contributions presented in this study are included in the article. Further inquiries can be directed to the corresponding authors.
